# The complete chloroplast genome of *Uncaria macrophylla* Wall. (Rubiaceae) and its phylogenetic analysis

**DOI:** 10.1080/23802359.2022.2075711

**Published:** 2022-05-13

**Authors:** Ni Zhang, Junrong Song, Hanfei Liu, Fei Li, Weidong Pan

**Affiliations:** a State Key Laboratory of Functions and Applications of Medicinal Plants, Guizhou Medical University, Guiyang, China; b The Key Laboratory of Chemistry for Natural Products of Guizhou Province and Chinese Academy of Sciences/Guizhou Provincal Engineering Research Center for Natural Drugs, Guiyang, China

**Keywords:** *Uncaria macrophylla* Wall., complete chloroplast genome, phylogenetic analysis

## Abstract

*Uncaria macrophylla* Wall. 1824 is one of the five original plants of Uncariae Ramulus cum Uncis, and its molecular genetic relationship with other four remains unclear. Here, we reported the complete chloroplast genome of *U. macrophylla*. The chloroplast genome is 155,145 bp in length, which includes paired inverted repeat regions of 25,665 bp, a large single-copy region of 85,758 bp and a small single-copy region of 18,057 bp. In total, 127 genes were predicted, including 81 protein-coding genes, 38 tRNA genes, and 8 rRNA genes. The phylogenetic analysis based on whole chloroplast genome sequences showed that *U. macrophylla* is closely related to *U. rhynchophylla*.

In the Pharmacopoeia of the People’s Republic of China, *Uncaria macrophylla* Wall. is one of the five original plants of Uncariae Ramulus cum Uncis (The State Pharmacopoeia Commission of China 2020). The dried stems with curved hooks of *U. macrophylla*, the traditional medicinal parts, have antihypertensive, analgesic, anticonvulsant, and associated symptoms (Kim et al. 2011; Liang et al. 2020; Qin et al. 2021). Rhynchophylline and isorhynchophylline have been confirmed as representative active components of *U. macrophylla* (Shi et al. 2003). In addition, based on 132 ITS sequences, the phylogenetic analysis of 12 species from *Uncaria* recorded in *Flora of China* was established (Li 2018). As a multiorigin traditional Chinese medicine, Uncariae Ramulus cum Uncis and its relative adulterants have been identified by the ITS2 barcode to ensure the accuracy and safety of clinical medication (Yao et al. 2019). The complete chloroplast genome is an important technology for the authentication of the botanical origin of Chinese medicines. It has not been reported for *U. macrophylla*. Therefore, it is necessary to generate complete chloroplast genomes to explore the origin and evolution of *U. macrophylla*.

Healthy and fresh samples were collected from Yunnan Branch of Institute of Medicinal Plants, Chinese Academy of Medical Sciences, Jinghong City, Xishuangbanna Prefecture, Yunnan Province (21°54′N, 101°04′E, 540 m above sea level). No specific permissions were required for any of the collection localities. The plants were identified by Prof. Deying Tang, Yunnan Branch of Institute of Medicinal Plants, Chinese Academy of Medical Sciences, Yunnan, China. A specimen was deposited at a local herbarium of the School of Life Sciences, Guizhou Normal University (https:sjxy.gznu.edu.cn, Zheng-wen Yu and yuzhengwen2001@126.com) under voucher number GZNUYZW202101025. Total genomic DNA (No. YX20210125901) was extracted using an E.Z.N. A Plant DNA kit (FEIYANG, Guangzhou, China) and stored in the biochemical laboratory (room number: 1403) of the School of Life Sciences, Guizhou Normal University. First, 1000 ng of DNA was used for the DNA sample preparations. Sequencing libraries were generated using the NEB NextV RUltra DNA Library Prep Kit for Illumina V R (NEB, Ipswich, MA). Total DNA was used to produce libraries with an average insert size of 400 bp. The library preparations were sequenced on an Illumina platform, after which 150 bp paired-end reads were generated. Filtered reads were assembled by employing the program GetOrganelle (Jin et al. 2019) with *U. rhynchophylla* as the initial reference genome (NC053701), and the assembled chloroplast genome was annotated by the online software GeSeq (Tillich et al. 2017). Ultimately, the complete chloroplast genome was submitted to GenBank with accession number MZ869757.

The length of the complete chloroplast genome sequence of *U. macrophylla* was 155,145 bp in size, consisting of a large single-copy (LSC, 85,758 bp) region, a small single-copy (SSC, 18,057 bp) region, and two inverted repeat (IRA and IRB) regions of 25,665 bp. In total, 127 genes were predicted, including 81 protein-coding genes (PCGs), 8 rRNAs, and 38 tRNAs. All rRNAs (*rrn16*, *rrn23*, *rrn4.5*, and *rrn5*), four PCGs (*rps7*, *rpl23*, *ndhB*, and *ycf2*), and seven tRNAs (*trnA-UGC*, *trnI-CAU*, *trnI-GAU*, *trnLCAA*, *trnN-GUU*, *trnR-ACG*, and *trnV-GAC*) were double copies among these assembled genes. One PCG (*rps12*) occurs in two copies. Intron–exon analysis showed that the majority (104 genes, 84%) of genes did not contain introns, whereas 20 (16%) genes contained introns.

To determine the phylogenetic position of *U. macrophylla*, 19 chloroplast genome sequences of Rubiaceae and 1 outgroup (*Lycoris chinensis*) were downloaded from GenBank to construct phylogenetic trees with *U. macrophylla* through the maximum-likelihood (ML) method. All sequences were aligned with MAFFT 7.409 (Katoh et al. 2002). The ML tree was performed using iqtree Version 1.6.12 with the TVM + F+R6 model based on 1000 bootstrap replicates. The phylogenetic tree indicated that *U. macrophylla* was resolved in a clade with *U. rhynchophylla*. (Figure 1).

**Figure 1.**
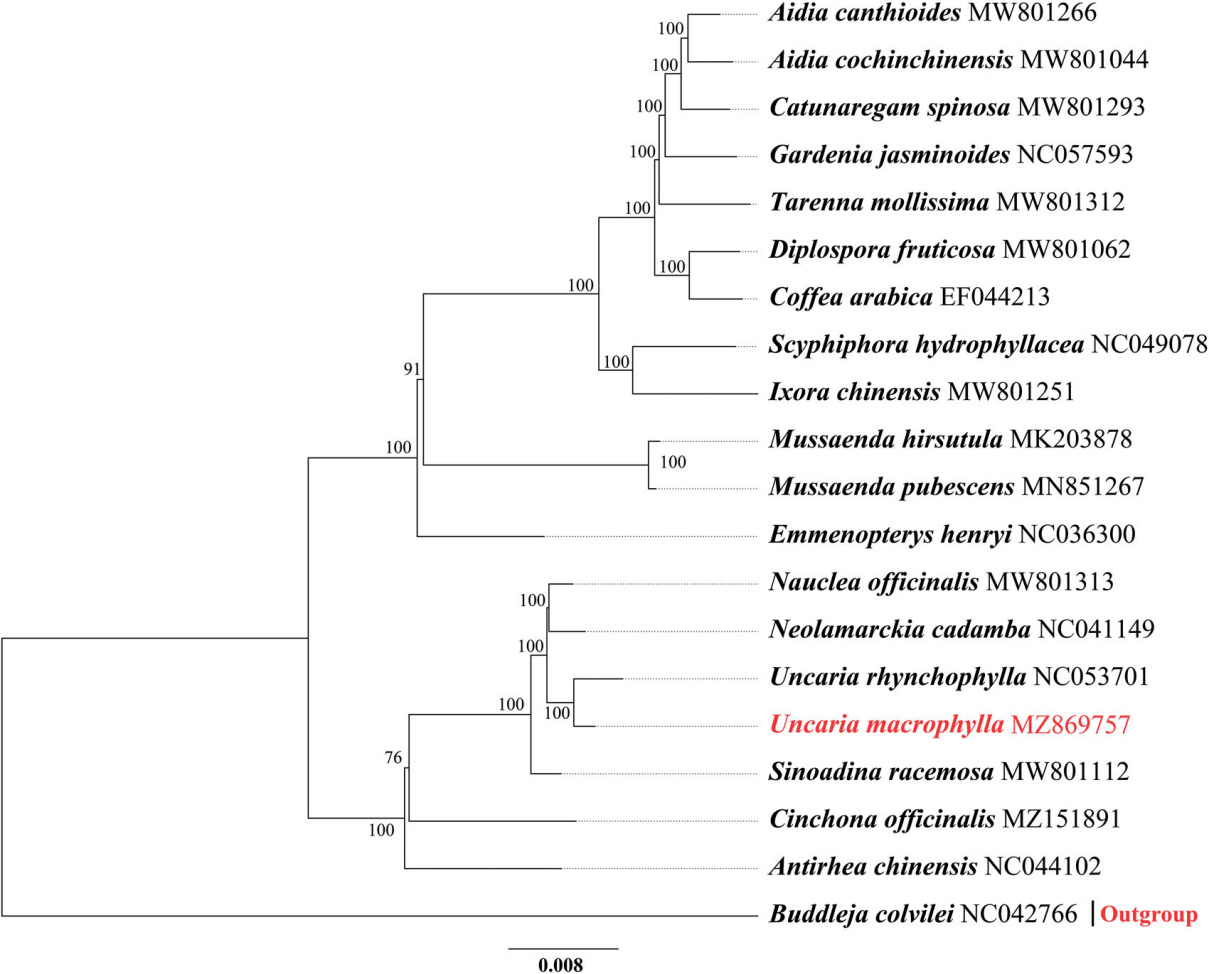
Maximum-likelihood tree based on the complete genome sequences of 20 species from the Gentianales. GenBank accession numbers are described. Bootstrap support values based on 1000 replicates are shown next to the nodes.

In conclusion, the *U. macrophylla* chloroplast genome sequence will provide useful data for further studies of *U. macrophylla* and contribute to understanding the phylogenetic relationships of the *Uncaria Schreber nom. Cons.* clade.

## Author contributions

Conceptualization, W.P.; Methodology, J.S. and H.L.; Writing—Original Draft Preparation, N.Z.; Writing—Review and Editing, F.L. and W.P.; and Funding acquisition, N.Z. All authors have read and agreed to the published version of the manuscript.

## Data Availability

The genome sequence data that support the findings of this study are openly available in GenBank of NCBI at https://www.ncbi.nlm.nih.gov, reference number MZ869757. The associated BioProject, SRA, and Bio-Sample numbers are PRJNA756526, SRR15540840, and SAMN20890004 respectively.
